# Asparaginase-like protein 1 and human endogenous retroviruses link immune and gene dysregulation in dementia

**DOI:** 10.3389/fcimb.2026.1777560

**Published:** 2026-03-25

**Authors:** Elena Rita Simula, Tommaso Ercoli, Elisa Ruiu, Milena Fais, Marta Noli, Paolo Solla, Leonardo Antonio Sechi

**Affiliations:** 1 Department of Biomedical Sciences, Microbiology, University of Sassari, Sassari, Italy; 2 Neurology Unit, University Hospital of Sassari, Sassari, Italy; 3 Microbiology and Virology, Azienda Ospedaliero-Universitaria (AOU) Sassari, Sassari, Italy

**Keywords:** Alzheimer’s disease, ASRGL1, dementia, gene expression, HERVs, humoral immune response

## Abstract

Dementia is increasingly recognized as a condition characterized not only by neurodegeneration but also by significant immune alterations, with potential consequences for both humoral immunity and peripheral gene regulation. However, the relationship between these processes when targeting neuronal and retroviral antigens remains poorly understood. This study investigated immune responses to asparaginase-like protein 1 and human endogenous retroviruses in different types of dementia. Plasma and peripheral blood mononuclear cells were collected from patients with dementia. Antibody reactivity against asparaginase-like protein 1 and human endogenous retroviruses was assayed by enzyme-linked immunosorbent assay, while peripheral gene expression was quantified by qPCR. Group comparisons and correlation analyses were performed. Patients exhibited increased antibody responses against asparaginase-like protein 1 and human endogenous retroviruses despite reduced peripheral expression of the corresponding genes. Within patients, antibodies against asparaginase-like protein 1 were inversely correlated with its transcript levels, whereas antibody responses to human endogenous retroviruses correlated positively with residual gene expression. Immune and transcriptional measures targeting these molecules were interrelated, indicating shared immune pathways. For the first time, this study identifies a coordinated relationship between humoral immune responses and peripheral gene expression in the dementia such as AD, MCI and mixed Dementia offering a novel context for interpreting immune dysregulation and suggesting potential immune-based biomarkers linked to neurodegenerative processes.

## Introduction

Dementia is a growing global health challenge, marked by a progressive decline in cognitive, behavioral, and functional abilities that gradually compromises everyday life (Yamasaki, 2026). As the population ages, the number of individuals affected by dementia continues to rise. Rather than a single disorder, dementia encompasses a broad spectrum of neurodegenerative and vascular conditions, often sharing overlapping pathological features (Thangaleela et al., 2025). In recent years, increasing attention has been directed toward the role of immune dysregulation, neuroinflammation, and systemic molecular changes in disease development and progression, underscoring the need for integrated approaches that combine peripheral and central biomarkers to better capture the biological complexity of dementia.

Alzheimer’s disease (AD) is the most common cause of dementia and is characterized by progressive cognitive decline, synaptic dysfunction, and widespread neurodegeneration. (Kamatham et al., 2024) Although the pathological hallmarks of AD have long been defined by the accumulation of amyloid-β plaques and neurofibrillary tangles, (Vanya et al., 2025) it is now widely recognized that these features alone do not fully account for disease onset, progression, or heterogeneity. (De Strooper and Karran, 2016) Increasing evidence supports a central role for immune dysregulation, both within the central nervous system (CNS) and at the systemic level, in shaping the course of the disease, with ongoing neurodegeneration promoting the release of neuronal and non-neuronal epitopes that can further broaden and sustain antigen-specific humoral immune responses (Britschgi et al., 2009; Heneka et al., 2015; Busse et al., 2017; Kocurova et al., 2022).

Mild cognitive impairment (MCI) represents an intermediate clinical stage between normal aging and dementia (Knopman and Petersen, 2014) Individuals with MCI exhibit measurable cognitive decline, most commonly affecting memory, while largely preserving functional independence (Bradfield, 2023). MCI is a heterogeneous condition, some patients progress to Alzheimer’s disease or other dementias, whereas others remain stable or even revert to normal cognition (Pandya et al., 2016). Mixed dementia refers to the coexistence of neurodegenerative pathology, most frequently Alzheimer-type changes, with cerebrovascular disease. Vascular contributions include ischemic lesions, small vessel disease, and cerebral hypoperfusion, which synergize with neurodegenerative processes to accelerate cognitive decline. (Sarhan et al., 2025) Clinically, mixed dementia often presents with executive dysfunction, attentional deficits, and stepwise progression, reflecting the combined impact of vascular and neurodegenerative damage (Sarhan et al., 2025). Other dementias include a group of less prevalent but clinically relevant neurodegenerative disorders, such as dementia with Lewy bodies, corticobasal degeneration, frontotemporal dementia and its association with motor neuron disease, as well as Amyloid Precursor Protein (APP)-related dementias (Dalla Barba and Boller, 1994). These conditions are characterized by distinct proteinopathies and clinical phenotypes, including α-synuclein or tau accumulation, early behavioral and language impairment, motor dysfunction, and, in some cases, early-onset neurodegeneration driven by amyloid pathology (Dalla Barba and Boller, 1994).

Neuroinflammation is a prominent and sustained component of dementia (Fuster-Matanzo et al., 2013; Kamila et al., 2025). Activated microglia and astrocytes, altered cytokine signaling, and chronic inflammatory stress contribute to neuronal damage and synaptic loss (Gao et al., 2023; Deng et al., 2024). Importantly, inflammatory processes are not confined to the CNS but extend to the peripheral immune compartment (Bettcher et al., 2021). Patients with dementia display systemic immune alterations, including changes in peripheral blood mononuclear cell (PBMC) composition, (Arosio et al., 2014; Amin et al., 2020; van Olst et al., 2024) features of immune-senescence, (Zhao et al., 2020) and immuno-metabolic remodeling (Wu et al., 2025). These peripheral immune changes are increasingly recognized as both reflective of, and contributory to, neurodegenerative processes (Trushina, 2019).

In parallel, growing attention has been directed toward the potential contribution of human endogenous retroviruses (HERVs) to autoimmune, cancer and, neurodegenerative disorders (Küry et al., 2018; Noli et al., 2022; Simula et al., 2023). HERVs are remnants of ancient retroviral infections integrated into the human genome and represent a substantial fraction of genomic content (Chen et al., 2025a; Simula et al., 2025). While most HERV sequences are transcriptionally silent under physiological conditions, specific families can be reactivated in response to inflammatory stimuli, cellular stress, or epigenetic dysregulation (Dopkins and Nixon, 2024). Among the different HERV families, HERV-W has emerged as a particularly immunologically active element. Its envelope protein has been shown to activate innate immune signaling pathways, including TLR4-mediated responses, leading to microglial activation and chronic neuroinflammation key pathogenic features of Alzheimer’s disease (Antony et al., 2011). In contrast, other HERV families, such as HERV-H, are more closely associated with transcriptional and epigenetic regulation and are generally considered less immunogenic. (Lu et al., 2014; Wang et al., 2014) HERV-H is characterized by high transcriptional activity (Gemmell et al., 2019) with HERV-H-derived transcripts known to modulate cellular states and gene expression programs, making it particularly relevant in the context of epigenetic and transcriptional dysregulation observed in Alzheimer’s disease (Lu et al., 2014; Wang et al., 2014; Göke et al., 2015; Zhang et al., 2019).

In the context of human endogenous retroviruses, it is important to consider their potential role as transcriptional regulators and their susceptibility to regulation by host mechanisms, including microRNA-mediated control, as reported in neurological conditions such as Parkinson’s disease (Simula et al., 2024). In fact, in amyotrophic lateral sclerosis (ALS), has been observed that HERV-K (HML-2) reactivation may interfere with the transcription of ASRGL1, leading to reduced ASRGL1 expression and subsequent accumulation of TDP-43, a pathological hallmark of the disease (Garcia-Montojo et al., 2024). Accordingly, in patients exhibiting HERV reactivation, a regulatory influence of these elements on host gene expression may occur and contribute to disease-related molecular alterations. Taken together, these observations support the rationale for examining both HERV-W and HERV-H in dementia, as they reflect complementary and interconnected aspects of HERV involvement, encompassing neuroinflammatory processes and transcriptional and epigenetic alterations.

Beyond retroviral elements, neurodegeneration is also associated with the pathological exposure of intracellular neuronal proteins that are normally non-immunogenic under physiological conditions. (Kocurova et al., 2022; Castro-Gomez and Heneka, 2024) Neuronal injury, oxidative stress, and blood-brain barrier dysfunction can promote the release of such proteins into extracellular and peripheral compartments, where they may become accessible to antigen-presenting cells. (Zhang et al., 2025) This process can lead to the development of autoantibody responses against neuronal or neuron-associated proteins, providing indirect markers of neuronal damage and disease burden.

In this context, ASRGL1 is a predominantly intracellular protein involved in amino acid metabolism and cellular homeostasis. (Cantor et al., 2009) Although it is not classically associated with immune function, neuronal damage may favor conditions that allow its abnormal exposure and subsequent immune recognition in pathological contexts. In this study, the detection of autoantibodies directed against ASRGL1 may therefore reflect ongoing or cumulative neurodegenerative processes rather than primary immune activation. At the same time, the expression of ASRGL1 in PBMCs may serve as an indicator of the functional and metabolic state of peripheral immune cells, which are known to undergo substantial remodeling in dementia (Arosio et al., 2014).

Despite increasing recognition of immune involvement in dementia, the relationship between humoral immune responses and peripheral gene expression remains poorly defined. In particular, it is unclear whether antibody responses against retroviral or neuronal antigens mirror peripheral transcriptional activity, reflect independent pathological processes, or converge on shared disease-related immune states.

In this study, we performed a combined analysis of humoral immune responses and peripheral gene expression in patients with dementia and healthy controls, focusing on two human endogenous retroviruses, HERV-W and HERV-H, and on the neuronal/metabolic protein ASRGL1. We aimed to investigate whether these biologically distinct targets reflect shared or distinct aspects of disease-associated immune remodeling, thereby contributing to a more comprehensive understanding of neuro-immune interactions in Alzheimer’s disease.

## Materials and methods

The study was conducted in accordance with the principles outlined in the Declaration of Helsinki. All participants provided written informed consent prior to inclusion in the study. The study protocol was reviewed and approved by the appropriate local Ethics Committee (Azienda Ospedaliero-Universitaria, Sassari, Italy; IRB number 2159/CE of the 17/03/2015), and all procedures involving human samples were performed in compliance with relevant institutional and regulatory guidelines.

### Samples

Peripheral whole blood samples were obtained from individuals with dementia and from healthy blood donors. A total of 94 patients, divided in AD (n=39), MCI (n=21), Mixed dementia (n=26) and, other dementias (comprising dementia with Lewy bodies, corticobasal degeneration, frontotemporal dementia, APP-related dementia) (n=8), were recruited between 2023 and 2025 (58 females and 36 males; median age = 77 years) at the Neurorehabilitation Service of the Azienda Ospedaliera Universitaria. A cohort of 46 healthy control subjects was enrolled during 2024 at the Blood Transfusion Centre of Sassari (25 females and 21 males; median age = 58 years). Detailed clinical and demographic characteristics of the study population are reported in **Supplementary Table 1**.

### Blood samples collection

Peripheral venous blood samples were obtained from the participants using K2-EDTA tubes. The collected whole blood was layered over an equal volume of Ficoll (Sigma-Aldrich, St. Louis, MO, USA) in a 15 mL tube and centrifuged for 20 min at 1800 RPM without brake. PBMCs were collected and stored at -80 °C. PBMCs were preserved in fetal bovine serum (FBS) containing 10% dimethyl sulfoxide (DMSO) for subsequent RNA extraction.

### RNA isolation and RT-qPCR analysis

Total RNA from PBMCs was purified using the RNeasy Mini Kit (QIAGEN, Milano, Italy) and treated with DNase to remove DNA contamination with the Turbo DNA-free kit (Thermofisher), following the manufacturer’s instructions. Then, the RNA concentration was measured with the Nanodrop (Thermofisher) and all the samples were adjusted to the same concentration. First-strand cDNA synthesis was performed using QuantiTect Reverse Transcription Kit (QIAGEN, Milano, Italy). No-RT (no Reverse Transcriptase) for each sample and no-TC (no Template Control) controls were made to check for DNA and reagent contamination, respectively. Subsequently, samples were analyzed by Real-Time-PCR using Quantinova SYBR Green PCR kit (QIAGEN, Milano, Italy), following the manufacturer’s instructions. The relative mRNA expression levels were calculated by the 2^-ΔΔCt^ method and HPRT1 mRNA levels were used for normalization. Gene-specific primer pairs are listed in **Table 1**.

**Table 1:** List of primers and epitope sequences used in the study.

Primers	Sequence
*HERV-W*	FW: GTATGTCTGATGGGGGTGGAG
	RV: CTAGTCCTTTGTAGGGGCTAGAG
*HERV-H*	FW: TTCACTCCATCCTTGGCTAT
	RV: CGTCGAGTATCTACGAGCAAT
*ASRGL1*	FW: AGTTCTTCCGAGGGCACAAG
	RV: TCCCACCTGATGAGAAACGC
*HPRT1*	FW: GCTATAAATTCTTTGCTGACCTGCTG
	RV: AATTACTTTTATGTCCCCTGTTGACTGG
Epitope	Sequence
*HERV-W_(248-262)_*	NSQCIRWVTPPTQIV
*HERV-H_(229-241)_*	LGRHLPCISLHPW
*ASRGL_(14-25)_*	PISKDRKERVHQ

The upper section reports the names and nucleotide sequences of the primers employed for gene expression analysis, while the lower section details the amino acid sequences of the epitopes used for antibody detection.

### Determination of antibodies by peptide ELISA

Epitope selection was performed using the Immune Epitope Database (IEDB - available at https://www.iedb.org - accessed August 2025), an online resource that integrates experimentally validated and computationally predicted immune epitopes. Candidate peptide sequences were selected based on predicted linear B-cell epitope characteristics, including antigenicity and surface accessibility, and were further refined to exclude sequence overlap with other known epitopic regions, thereby ensuring specificity of the antibody detection. Selected epitopes were synthesized at >95% purity (LifeTein, South Plainfield, NJ 07080, USA) and dissolved in dimethyl sulfoxide (DMSO) to obtain 10 mM stock solutions. The list of epitopes is reported in **Table 1**.

The indirect enzyme-linked immunosorbent assay (ELISA) was performed to detect antibody (Ab) reactivity against the selected peptides. Ninety-six-well microplates (Nunc) were coated overnight at 4 °C with 3 µM peptide diluted in 0.05 M carbonate-bicarbonate buffer (pH 9.5; Sigma). After coating, plates were blocked with 1% skimmed milk (Sigma) for 1 hour at 37 °C to prevent nonspecific binding and subsequently washed twice with Tris-buffered saline containing 0.05% Tween-20 (TBS-T). Plasma samples were diluted 1:20 in 1% skimmed milk prepared in TBS-T, and 100 µL of each diluted sample were added to the wells. Plates were incubated for 2 hours at 37 °C. Following incubation, plates were washed with TBS-T and incubated for 1 hour at 37 °C with an alkaline phosphatase-conjugated goat anti-human immunoglobulin G (IgG) polyclonal antibody (1:1000; Sigma). After four additional washes with TBS-T, plates were incubated with para-nitrophenylphosphate substrate (Sigma) for 15 minutes at room temperature in the dark. Absorbance was measured at 405 nm using a microplate reader (Molecular Devices).

Each plasma sample was analyzed in triplicate, and the results represent the mean of two independent experiments performed under identical conditions. Blank values, wells coated with peptides and incubated with the secondary antibody alone, were subtracted from all sample readings. Positive and negative control plasma samples were included in each experiment. A reference plasma sample was included as a calibrator on each plate to minimize inter-plate variability. Results are expressed as the mean optical density (OD) values of triplicate measurements.

### Statistical analysis

ELISA optical density (OD) values were analyzed using non-parametric statistics due to their non-normal distribution. Differences in antibody reactivity between patients with dementia and healthy controls were assessed using the Mann-Whitney U test. Antibody positivity thresholds were determined by receiver operating characteristic (ROC) curve analysis, and group differences in seropositivity were subsequently evaluated using Fisher’s exact test.

Quantitative PCR (qPCR) data were analyzed using the comparative ΔΔCt formula, with relative expression levels calculated as fold change values using the 2^-ΔΔCt^ method. Given the non-Gaussian distribution of qPCR-derived data, group comparisons were performed using the Mann-Whitney U test. Correlation analyses were conducted using Spearman’s rank correlation coefficient. Statistical significance was defined as a p-value < 0.05.

## Results

**Figure 1:**
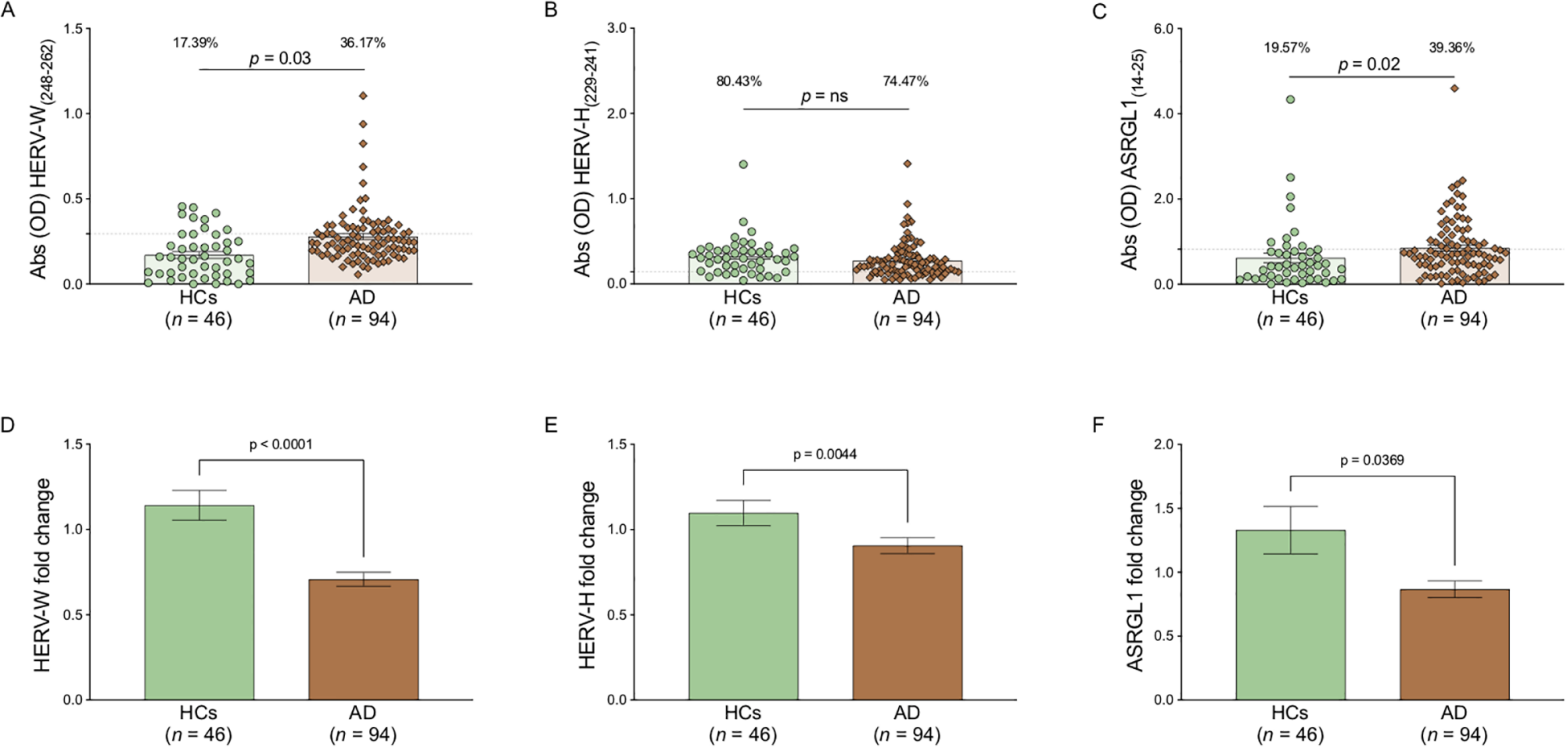
ELISA-based analysis of antibody reactivity against epitopes derived from HERV-W, HERV-H, and ASRGL1 **(A-C).** Plasma samples from patients with dementia and healthy controls (HC) were analyzed using an indirect ELISA. Results are expressed as median ± standard error of the mean (SEM). Mann-Whitney p-values and the percentage of antibody-positive individuals, determined by Fisher’s exact test, are reported in the upper part of each graph. Positivity thresholds were established by receiver operating characteristic (ROC) curve analysis. Gene expression analysis of HERV-W, HERV-H, and ASRGL1 in PBMCs from patients with dementia and HC subjects **(D-F).** Relative gene expression was assessed by quantitative PCR (qPCR) and is reported as median ± SEM of fold change values calculated using the 2^-ΔΔCt^ method. Mann-Whitney p-values are indicated in the upper part of each graph. Statistical significance was defined as p < 0.05.

We analyzed antibody responses directed against epitopes derived from HERV-W, ASRGL1, and HERV-H proteins in patients with dementia and healthy controls (HC) (**Figure 1**). This analysis revealed a significantly higher humoral response in patients compared with controls for HERV-W (Fisher’s exact test, *p* = 0.03; positivity rate: HC 17.39%, AD 36.17%) (**Figure 1A**) and ASRGL1 (Fisher’s exact test, *p* = 0.02; positivity rate: HC 19.57%, AD 39.36%) (**Figure 1C**). In contrast, a lower proportion of antibody-positive individuals was observed in patients compared with controls for HERV-H, although this difference did not reach statistical significance (Fisher’s exact test, *p* = 0.527; positivity rate: HC 80.43%, AD 74.47%) (**Figure 1B**).

In parallel, gene expression analysis performed on PBMCs showed that patients had significantly lower expression levels of all three genes examined compared with healthy controls: HERV-W (*p* < 0.0001) (**Figure 1D**), HERV-H (*p* = 0.004) (**Figure 1E**), and ASRGL1 (*p* = 0.036) (**Figure 1F**). Although this pattern may appear discordant when considered alongside the antibody data, it is consistent when interpreted within the pathological context of the disease and prompted further investigation of the relationship between humoral responses and gene expression through correlation analyses.

**Figure 2:**
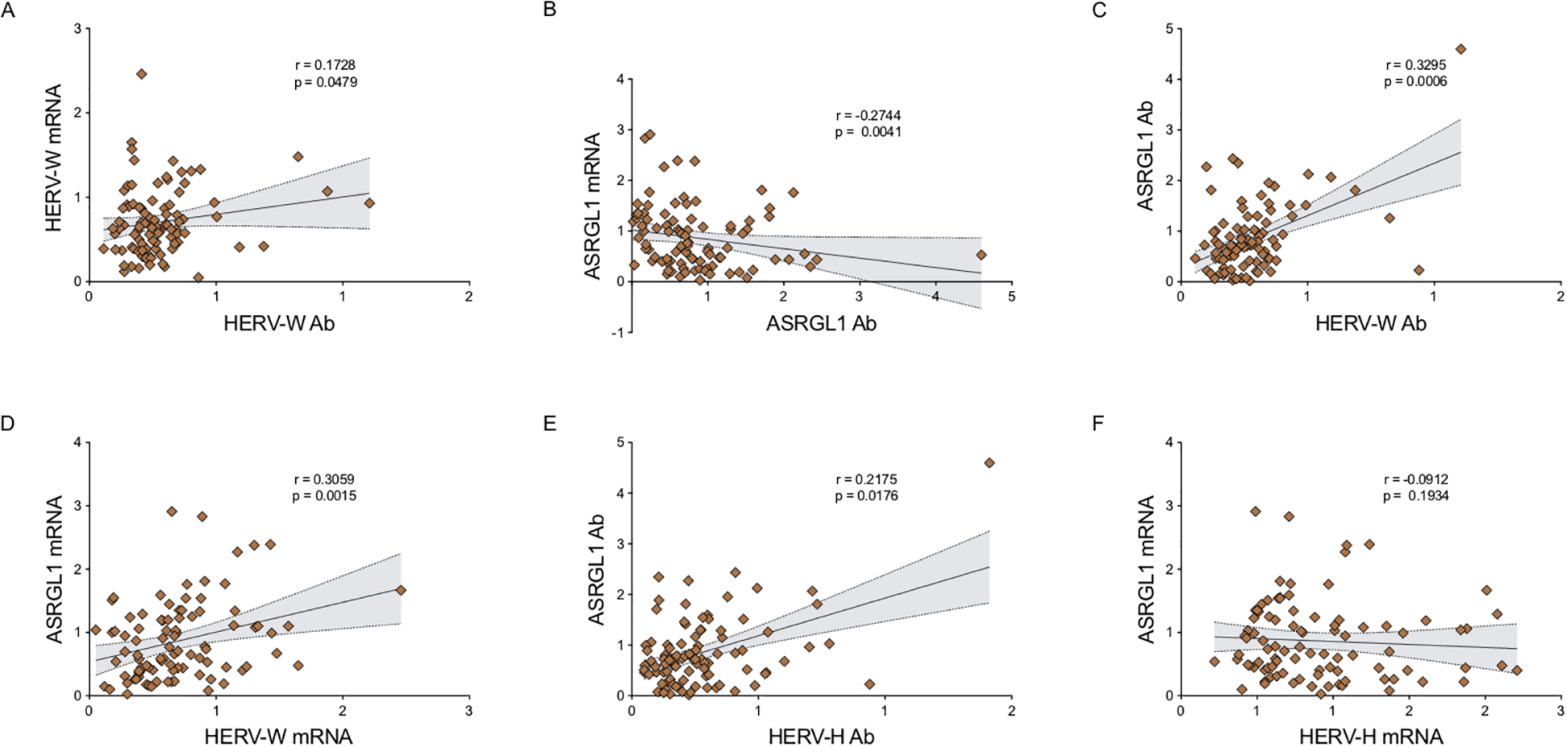
Correlation analyses among gene expression levels and antibody responses in patients withdementia **(A-F)**. Correlation analyses between gene expression levels and antibody responses for the same targets, as well as between gene expression and antibody levels across different targets. Scatter plots display the distribution of samples. The black line represents the fitted regression line, while the shaded area indicates the 95% confidence interval. For each correlation, Spearman’s rank correlation coefficient (r) and the corresponding p-value are reported. Statistical significance was defined as p < 0.05.

Correlation analyses conducted within the patient group revealed several significant associations (**Figure 2**). A positive correlation was observed between HERV-W Ab levels and HERV-W gene expression (*r* = 0.1728; *p* = 0.0479) (**Figure 2A**). In addition, ASRGL1 Ab levels correlated positively with HERV-W Ab levels (*r* = 0.3295; *p* = 0.0006) (**Figure 2C**) and ASRGL1 gene expression showed a positive correlation with HERV-W gene expression (*r* = 0.3059; *p* = 0.0015) (**Figure 2D**). A further positive correlation was detected between ASRGL1 and HERV-H Ab levels (*r* = 0.2175; *p* = 0.0176) (**Figure 2E**).

Notably, ASRGL1 gene expression was inversely correlated with ASRGL1 Ab levels (*r* = -0.2744; *p* = 0.0041) (**Figure 2B**). In contrast, the correlation between ASRGL1 gene expression and HERV-H gene expression, while negative, did not reach statistical significance (*r* = -0.0912; *p* = 0.1934) (**Figure 2F**).

**Figure 3:**
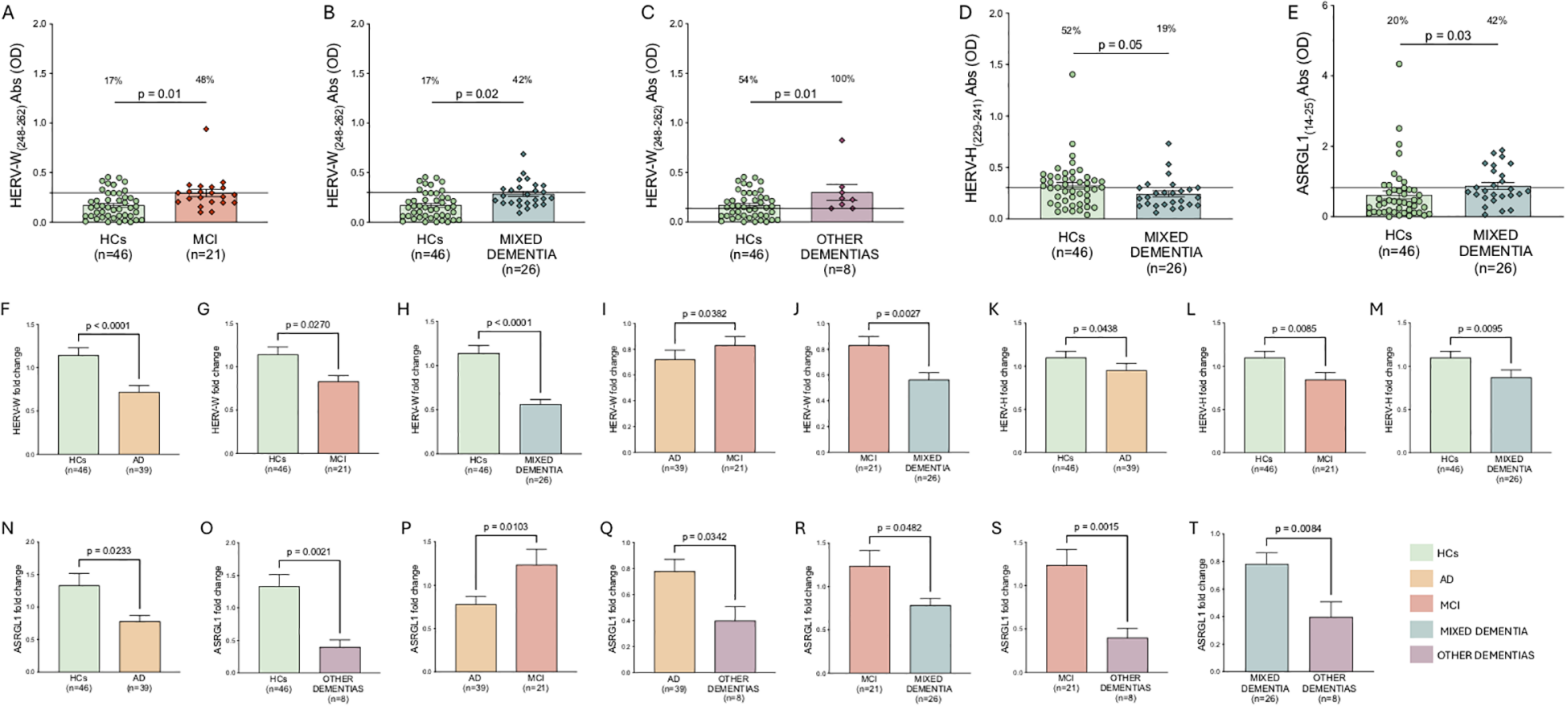
ELISA-based analysis of antibody reactivity against epitopes derived from HERV-W, HERV-H, and ASRGL1 **(A-E).** Plasma samples from patients with dementia and healthy controls (HC) were analyzed using an indirect ELISA. Results are expressed as median ± standard error of the mean (SEM). Mann-Whitney p-values and the percentage of antibody-positive individuals, determined by Fisher’s exact test, are reported in the upper part of each graph. Positivity thresholds were established by receiver operating characteristic (ROC) curve analysis. Gene expression analysis of HERV-W, HERV-H, and ASRGL1 in PBMCs from patients with dementia and HC subjects **(F-T).** Relative gene expression was assessed by quantitative PCR (qPCR) and is reported as median ± SEM of fold change values calculated using the 2^-ΔΔCt^ method. Mann-Whitney p-values are indicated in the upper part of each graph. Statistical significance was defined as p < 0.05.

We further performed subgroup analyses by stratifying patients with dementia according to diagnostic category and comparing each subgroup both with healthy controls and with the other dementia subgroups (**Figure 3**). For HERV-W_(248-262),_ patients with MCI (**Figure 3A**), mixed dementia (**Figure 3B**), and other dementias (**Figure 3C**) showed significantly higher antibody levels compared with healthy controls. Specifically, seropositivity was observed in 48% of MCI patients versus 17% of healthy controls (p = 0.01), in 42% of patients with mixed dementia versus 17% of healthy controls (p = 0.02), and in 100% of patients with other dementias versus 54% of healthy controls (p = 0.01). Regarding HERV-W gene expression, significant differences were observed among the analyzed groups. Specifically, an increased HERV-W expression was detected in HC individuals compared with AD patients (p < 0.0001) (**Figure 3F**), MCI (p = 0.02) (**Figure 3G**) and in those with mixed dementia (p < 0.0001) (**Figure 3H**). In addition, higher gene expression levels were observed in MCI patients compared to AD patients (p = 0.03) (**Figure 3I**) and patients with mixed dementia (p = 0.002) (**Figure 3J**).

Regarding HERV-H_(229-241),_ patients with mixed dementia showed significantly lower antibody levels compared with healthy controls. Specifically, seropositivity was observed in 19% of mixed dementia patients versus 52% of healthy controls (p = 0.05) (**Figure 3D**). HERV-H gene expression was significantly downregulated in AD, MCI, and mixed dementia compared with healthy controls (p = 0.04, p = 0.008, and p = 0.009, respectively) (**Figures 3K–M**).

Antibodies against ASRGL1_(14-25)_ were increased exclusively in the mixed dementia group compared to healthy controls, with seropositivity observed in 42% of patients versus 20% of controls (p = 0.03) (**Figure 3E**). With respect to gene expression, ASRGL1 was significantly downregulated in patients with AD and other dementias compared with healthy controls (p = 0.02 and p = 0.002, respectively; **Figure 3**, panels N and O). MCI patients showed significantly higher ASRGL1 expression compared with AD, mixed dementia, and other dementias (p = 0.01, p = 0.04, and p = 0.001, respectively; **Figures 3P, R, S**). Finally, patients with other dementias exhibited lower ASRGL1 expression compared with both AD and mixed dementia (p = 0.03 and p = 0.008, respectively; **Figures 3Q, T**).

No statistically significant differences were observed with respect to sex, either when considering the overall cohort of patients with dementia or when patients were further stratified by dementia subtype (S7-S12).

**Figure 4:**
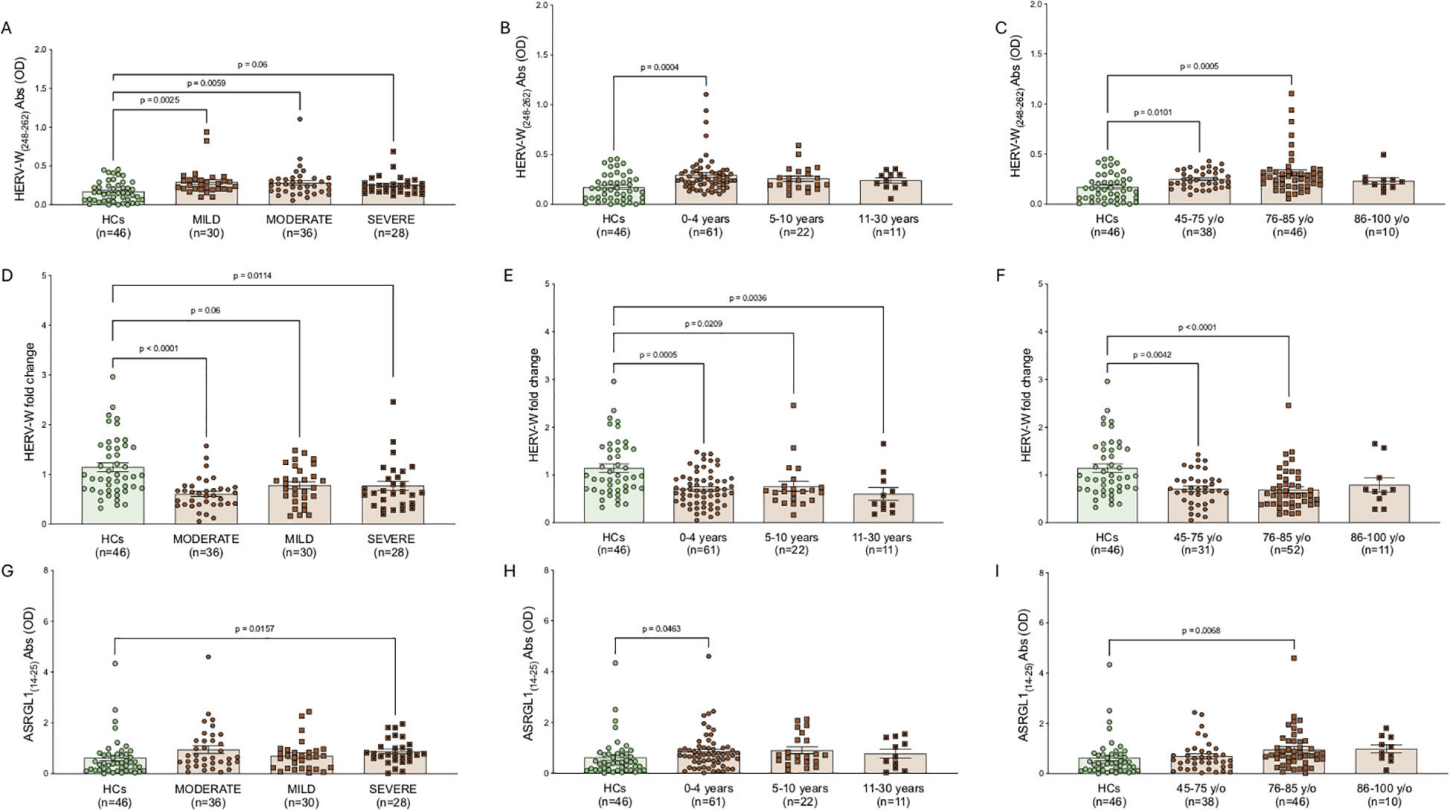
Analysis of antibody levels and gene expression of HERV-W and ASRGL1 in patients with dementia stratified by disease severity, disease duration, and age. Panels show statistical comparisons performed using the Kruskal-Wallis test. Specifically, panels report HERV-W antibody levels stratified by disease severity **(A)**, disease duration **(B)**, and age **(C)**; HERV-W gene expression stratified by disease severity **(D)**, disease duration **(E)**, and age **(F)**; and ASRGL1 antibody levels stratified by disease severity **(G)**, disease duration **(H)**, and age **(I)**. Statistically significant p values are reported above each comparison. The legend indicates the color coding corresponding to the different dementia subgroups.

With respect to disease severity, severity was assessed in a cross-sectional manner, independently of dementia subtype (**Figure 4**). For HERV-W_(248-262)_, our analyses revealed statistically significant differences between healthy controls and patients with mild disease (p = 0.0025) (**Figure 4A**), as well as between healthy controls and patients with moderate disease (p = 0.0059)(**Figure 4A**). HERV-W gene expression levels showed a significant downregulation in patients compared with healthy controls, specifically in the moderate (p < 0.0001) (**Figure 4D**) and severe (p = 0.011) (**Figure 4D**) disease severity groups.

For HERV-H, no statistically significant differences were detected in either humoral immune responses or gene expression levels.

For ASRGL1, no statistically significant differences were observed in gene expression or antibody levels, with the exception of a difference between healthy controls and patients with severe disease, in whom higher antibody levels were detected compared with controls (p = 0.015) (**Figure 4G**).

Regarding the disease duration (**Figure 4**), HERV-W antibodies resulted higher in patients with a disease duration of 0–4 years (p=0.0004) (**Figure 4B**); healthy controls exhibited significantly higher HERV-W gene expression compared with patients with a disease duration of 0–4 years (p = 0.0005), 5–10 years (p = 0.02), and 11–30 years (p = 0.003) (**Figure 4E**). In addition, an interesting finding was that antibody levels against ASRGL1_(14-25)_ were markedly higher in patients with a disease duration of 0–4 years compared with healthy controls (p = 0.04) (**Figure 4H**).

In the context of age stratification, patients with dementia were divided into three age groups (45–75 years, 76–85 years, and 86–100 years) (**Figure 4**). For HERV-W, patients aged 45–75 and 76–85 years exhibited significantly higher antibody levels compared with healthy controls (p = 0.01 and p = 0.0005, respectively) (**Figure 4C**). Similar differences were observed for HERV-W gene expression, with lower expression levels in patients compared with healthy controls (p = 0.004 for the 45–75 group and p < 0.0001 for the 76–85 group) (**Figure 4F**). In addition, in the context of ASRGL1, antibody levels were significantly higher in patients aged 76–85 years compared with healthy controls (p = 0.006) (**Figure 4I**).

The corresponding data are presented in **Figures 3**, **4** and in the **Supplementary Materials** (**Supplementary Figure 1-S20**).

## Discussion

In this study, for the first time, we investigated humoral immune responses and peripheral gene expression profiles directed against biologically distinct targets, two human endogenous retroviruses (HERV-W and HERV-H) and a neuronal/metabolic protein (ASRGL1), in patients with dementia compared with healthy controls. By group-level comparisons and correlation analyses within the patient cohort, our findings delineate an immune-transcriptional framework characterized by coherent alterations in peripheral immune status, target-specific humoral activation, and structured inter-individual variability.

A common feature across all genes examined was their reduced expression in PBMCs from patients relative to HCs suggesting the presence of a globally altered transcriptional state within the peripheral immune compartment of individuals with dementia. Growing evidence indicates that dementia is associated with systemic immune alterations, including changes in PBMC subset composition, features of immunosenescence, chronic low-grade inflammation, and immune-metabolic reprogramming. (Arosio et al., 2014; Chen et al., 2025b) Within this context, the coordinated downregulation of genes belonging to distinct functional categories, ranging from metabolic enzymes and endogenous retroelements, likely reflects a global shift in immune cell functional state rather than isolated, gene-specific dysregulation.

Against this shared transcriptional background, Ab profiles provide an additional and more discriminative layer of information that is strongly target-dependent. For the first time, we demonstrate that ASRGL1-specific autoantibodies are significantly elevated in patients despite reduced ASRGL1 expression in PBMCs. ASRGL1 is a predominantly intracellular protein involved in amino acid metabolism and is not expected to be immunogenic under physiological conditions. (Cantor et al., 2009) Its recognition by the adaptive immune system is therefore plausibly explained by pathological exposure following neuronal injury. Neurodegeneration, together with oxidative stress and blood-brain barrier dysfunction, may facilitate the release of intracellular neuronal proteins into extracellular and peripheral compartments, enabling antigen presentation and the development of sustained humoral immune responses.

Importantly, within the dementia group, ASRGL1 Ab levels were inversely correlated with ASRGL1 transcript abundance in PBMCs. This negative correlation indicates that stronger humoral immune responses against ASRGL1 are associated with a more pronounced reduction of ASRGL1 expression in peripheral immune cells. Such a pattern is consistent with a damage-dysfunction axis, in which increased neuronal injury leads to greater antigen release and antibody production, while simultaneously contributing to systemic immune and metabolic alterations that suppress gene expression in PBMCs. In addition, ASRGL1 downregulation may impair neuronal proteostasis, exacerbating protein aggregation in dementia. Future studies should investigate the relationship between ASRGL1 expression and the accumulation of amyloid-β and intracellular neurofibrillary tangles within the central nervous system of patients affected by dementia, with particular emphasis on Alzheimer’s disease.

Previous studies have consistently highlighted ASRGL1 as a gene of relevance in Alzheimer’s disease, although investigated within different experimental and biological frameworks. ASRGL1 has been analyzed primarily in the context of brain transcriptomics, where its alteration was linked as reflecting neuronal and metabolic dysfunction associated with neurodegeneration, without a focus on immune mechanisms or on compartmental differences. (Xing et al., 2025) More recently, ASRGL1 has been identified as a hub gene within glutamine metabolism-related signatures using machine learning approaches applied to bulk transcriptomic datasets, emphasizing its diagnostic performance across cohorts. In this setting, differences in ASRGL1 expression likely reflect the use of heterogeneous bulk samples and the influence of cellular composition, rather than cell-type-specific regulatory processes (Vardarajan et al., 2018).

A distinct yet complementary pattern emerged from the analysis of HERV-W. Patients exhibited significantly elevated levels of anti-HERV-W antibodies alongside reduced HERV-W expression in PBMCs compared with healthy controls. HERV-W is a tightly regulated endogenous retroelement whose expression is normally suppressed through epigenetic and immune mechanisms but can be transiently reactivated in response to inflammation and cellular stress, particularly within the central nervous system and glial cells. (Kassiotis and Stoye, 2016) Proteins derived from HERV-W, especially envelope-related products, are highly immunogenic and capable of eliciting robust innate and adaptive immune responses (Rangel et al., 2022).

Within the patient cohort, anti-HERV-W Ab levels positively correlated with HERV-W transcript abundance in PBMCs indicating that, despite overall transcriptional repression, inter-individual variability in residual peripheral HERV-W expression remains biologically coupled to humoral immune activation. Patients with relatively higher HERV-W expression may therefore represent individuals in whom transcriptional control of the retroelement is less efficient or more dynamically regulated, resulting in greater cumulative antigen exposure and stronger Ab responses. This positive correlation supports a model in which systemic repression of HERV-W coexists with incomplete or heterogeneous control, allowing residual expression to retain immunological relevance.

An additional observation of particular relevance is the positive correlation between HERV-W and ASRGL1, detected both at the Ab level and at the level of gene expression within the patient group. The positive correlation between anti-HERV-W and anti-ASRGL1 Abs indicates that humoral responses against these two distinct targets tend to co-occur in the same individuals, suggesting a shared pathological context characterized by enhanced neurodegeneration and immune activation. Similarly, the positive correlation between HERV-W and ASRGL1 transcript levels in PBMCs suggests that both genes are sensitive to a common peripheral immune or metabolic state, with relatively higher expression in patients with more preserved immune competence and lower expression in those with more advanced systemic dysfunction.

In this context, it is plausible to hypothesize that HERV-W reactivation may indirectly contribute to ASRGL1-related alterations by amplifying neuroinflammatory processes and neuronal stress. Rather than implying a direct regulatory interaction, HERV-W may act upstream by exacerbating pathological conditions that favor neuronal damage and the release of intracellular antigens, including ASRGL1. A conceptually similar hypothesis has been proposed in the context of ALS, where a functional link between HERV-K activity and ASRGL1 modulation has been suggested, supporting the notion that endogenous retroelements may contribute, under specific pathological conditions, to the regulation of neuronal or neuron-associated genes (Garcia-Montojo et al., 2024).

In support of this hypothesis, it is noteworthy that the ASRGL1 locus harbors multiple LTR elements attributable to the HERV-W family, characterized by a heterogeneous genomic architecture. Specifically, LTR66 is oriented in the same direction as the gene and may potentially contribute to transcriptional regulation in a promoter-like manner, whereas MER4D1, LTR10C, and LTR8A are oriented in antisense. Antisense-oriented LTRs are known to exert regulatory effects through enhancer-like activity, chromatin remodeling, or the generation of antisense transcripts. (Garcia-Montojo et al., 2024) Taken together, this supports the possibility of a complex regulatory influence of HERV-W-derived elements on ASRGL1 expression, although its functional relevance requires dedicated experimental validation.

By contrast, HERV-H displayed a different immunological and transcriptional profile. Although HERV-H expression was reduced in PBMCs from patients compared with controls, antibody levels directed against HERV-H did not differ significantly between groups. Nevertheless, within the patient cohort, anti-HERV-H antibody levels positively correlated with anti-ASRGL1 antibodies, indicating that humoral responses against HERV-H tend to co-occur with those directed against neuronal antigens in patients with higher pathological burden. This suggests that HERV-H participates in the broader humoral activation field as a context-dependent or secondary immunological target. At the transcriptional level, however, the relationship between HERV-H and ASRGL1 differed markedly. The correlation between HERV-H and ASRGL1 gene expression in PBMCs was negative but not statistically significant, indicating the absence of robust functional connection in their peripheral regulation. This dissociation suggests that, unlike HERV-W, HERV-H is not integrated into the shared peripheral transcriptional field associated with ASRGL1 expression. Given the established role of HERV-H in transcriptional and epigenetic regulation rather than in the production of strongly immunogenic proteins, (Sexton et al., 2022) this pattern is consistent with a model in which HERV-H contributes to humoral immune activation indirectly, without acting as a driver of peripheral immune cell transcriptional state.

Overall, our subgroup analyses highlight several relationship between humoral immune responses and peripheral gene expression across the different types of dementia. In particular, HERV-W emerged as a constant marker, characterized by increased antibody responses in multiple dementia subtypes and disease stages, coupled with a consistent downregulation of peripheral gene expression.

Notably, patients with MCI displayed a distinct molecular profile, with higher gene expression levels of HERV-W and ASRGL1 compared with AD and mixed dementia, along with an enhanced humoral responses compared to the HCs.

In contrast, ASRGL1-related immune responses appeared more context-dependent, emerging selectively in mixed dementia, severe disease, and early disease duration, suggesting involvement in specific pathological or metabolic processes.

Overall, this study contributes to a deeper understanding of humoral immune responses and peripheral gene expression in dementia, highlighting how distinct molecular targets capture different yet interconnected facets of a shared pathological framework. At the same time, one limitation of our study concerns the control population, which was younger than the patient cohort. This reflects the difficulty in recruiting age-matched healthy controls, as blood donation is often not permitted for elderly individuals, even when they are in good health. Consequently, unless blood sampling is performed for clinical reasons, obtaining biological samples from individuals of comparable age remains challenging. However, the coordinated attenuation of gene expression in PBMCs, together with selective and structured humoral responses, supports a target-dependent model of neuro-immune interaction. Importantly, the integration of plasma and transcriptional immune parameters highlights features that may have relevance as candidate immune-based biomarkers, offering understanding into the contribution of peripheral immune alterations and endogenous retroelements to neurodegenerative disorders.

## Data Availability

The raw data supporting the conclusions of this article will be made available by the authors, without undue reservation.
