# Supplementary material for: Asparaginase-like protein 1 and human endogenous retroviruses link immune and gene dysregulation in dementia
Source: Front Cell Infect Microbiol. 2026 Mar 25;16:1777560. doi: 10.3389/fcimb.2026.1777560 (PMC13058804; doi:10.3389/fcimb.2026.1777560)
Supplement: Supplementary file 1 [file Table1.docx]

Supplementary materials

Asparaginase-like Protein 1 and Human Endogenous Retroviruses

**Link Immune and Gene Dysregulation in Dementia**

Elena Rita Simula^1^, Tommaso Ercoli^2^, Elisa Ruiu^2^, Milena Fais^1^, Marta Noli^1^, Paolo Solla^2^, Leonardo Antonio Sechi^1,3*^.

^1^ Department of Biomedical Sciences, Microbiology, University of Sassari, Sassari, 07100, Italy.

^2^ Neurological Unit, AOU Sassari, Sassari, 07100, Italy.

^3^ Microbiology and Virology, AOU Sassari, 07100, Italy.

*** Correspondence:**
sechila@uniss.it

Keywords: Alzheimer’s disease, Dementia, ASRGL1, HERVs, Humoral immune response, Gene expression.

| DEMENTIA types | N | Sex  (F/M) | Age, years  (median) | Disease severity  (Mild / Moderate / Severe) |
| --- | --- | --- | --- | --- |
| Alzheimer’s disease (AD)* | 39 | 24 / 15 | 78 | 4 / 22 / 13 |
| Mild cognitive impairment (MCI) | 21 | 16 / 5 | 75 | 20 / 1 / 0 |
| Mixed / vascular dementia | 26 | 14 / 12 | 76 | 5 / 10 / 11 |
| Other dementias† | 8 | 4 / 4 | 77 | 1 / 3 / 4 |

**Table S1. Clinical distribution of patients across the dementia spectrum.** Diagnostic categories are reported together with the number of subjects (N), sex distribution (F/M), median age, and disease severity stratified as mild, moderate, or severe.

**Figure S1**. **Analysis of antibody levels against HERV-W_(248-262)_ in patients with dementia stratified into clinical subgroups.** Panels A-J report pairwise comparisons conducted using the Mann-Whitney U test. When appropriate, differences in categorical distributions were further evaluated using Fisher’s exact test. *P* values are reported above each comparison. Percentage values indicate the proportion of seropositive individuals relative to the corresponding reference group. The dashed line indicates, where applicable, the cutoff value determined by receiver operating characteristic (ROC) curve analysis, selected to maintain a test specificity above 80%. Statistical significance was defined as p < 0.05.

**Figure S2.** **Quantitative PCR (qPCR) analysis of HERV-W gene expression in patients with dementia stratified into clinical subgroups**. Panel A shows the statistical comparison performed using the Kruskal-Wallis test, whereas panels B-K report pairwise comparisons conducted using the Mann-Whitney U test. When appropriate, differences in categorical distributions were further evaluated using Fisher’s exact test. *P* values are reported above each comparison. Statistical significance was defined as p < 0.05.

**Figure S3**. **Analysis of antibody levels against HERV-H_(229-241)_ in patients with dementia stratified into clinical subgroups.** Panela A-J report pairwise comparisons conducted using the Mann-Whitney U test. When appropriate, differences in categorical distributions were further evaluated using Fisher’s exact test. *P* values are reported above each comparison. Percentage values indicate the proportion of seropositive individuals relative to the corresponding reference group. The dashed line indicates, where applicable, the cutoff value determined by receiver operating characteristic (ROC) curve analysis, selected to maintain a test specificity above 80%. Statistical significance was defined as p < 0.05.

**Figure S4.** **Quantitative PCR (qPCR) analysis of HERV-H gene expression in patients with dementia stratified into clinical subgroups**. Panela A-J report pairwise comparisons conducted using the Mann-Whitney U test. When appropriate, differences in categorical distributions were further evaluated using Fisher’s exact test. *P* values are reported above each comparison. Statistical significance was defined as p < 0.05.

**Figure S5**. **Analysis of antibody levels against ASRGL1_(14-25)_ in patients with dementia stratified into clinical subgroups.** Panel A shows the statistical comparison performed using the Kruskal-Wallis test, whereas panels B-K report pairwise comparisons conducted using the Mann-Whitney U test. When appropriate, differences in categorical distributions were further evaluated using Fisher’s exact test. *P* values are reported above each comparison. Percentage values indicate the proportion of seropositive individuals relative to the corresponding reference group. The dashed line indicates, where applicable, the cutoff value determined by receiver operating characteristic (ROC) curve analysis, selected to maintain a test specificity above 80%. Statistical significance was defined as p < 0.05.

**Figure S6.** **Quantitative PCR (qPCR) analysis of ASRGL1 gene expression in patients with dementia stratified into clinical subgroups**. Panels A-J report pairwise comparisons conducted using the Mann-Whitney U test. When appropriate, differences in categorical distributions were further evaluated using Fisher’s exact test. *P* values are reported above each comparison. Statistical significance was defined as p < 0.05.

**Figure S7. Analysis of antibody levels against HERV-W_(248-262)_ in patients with dementia, stratified by sex.** Panels show pairwise comparisons performed using the Mann-Whitney U test. *P* values are reported above each comparison. The legend indicates the color coding corresponding to the different dementia subgroups. Statistical significance was defined as p < 0.05.

**Figure S8. Quantitative PCR (qPCR) analysis of HERV-W gene expression in patients with dementia, stratified by sex.** Panels show pairwise comparisons performed using the Mann-Whitney U test. *P* values are reported above each comparison. The legend indicates the color coding corresponding to the different dementia subgroups. Statistical significance was defined as p < 0.05.

**Figure S9. Analysis of antibody levels against HERV-H_(229-241)_ in patients with dementia, stratified by sex.** Panels show pairwise comparisons performed using the Mann-Whitney U test. *P* values are reported above each comparison. The legend indicates the color coding corresponding to the different dementia subgroups. Statistical significance was defined as p < 0.05.

**Figure S10. Quantitative PCR (qPCR) analysis of HERV-H gene expression in patients with dementia, stratified by sex.** Panels show pairwise comparisons performed using the Mann-Whitney U test. *P* values are reported above each comparison. The legend indicates the color coding corresponding to the different dementia subgroups. Statistical significance was defined as p < 0.05.

**Figure S11. Analysis of antibody levels against ASRGL1_(14-25)_ in patients with dementia, stratified by sex.** Panels show pairwise comparisons performed using the Mann-Whitney U test. *P* values are reported above each comparison. The legend indicates the color coding corresponding to the different dementia subgroups. Statistical significance was defined as p < 0.05.

**Figure S12. Quantitative PCR (qPCR) analysis of ASRGL1 gene expression in patients with dementia, stratified by sex.** Panels show pairwise comparisons performed using the Mann-Whitney U test. *P* values are reported above each comparison. The legend indicates the color coding corresponding to the different dementia subgroups. Statistical significance was defined as p < 0.05.

**Figure S13**. **Analysis of antibody levels against HERV-W_(248-262)_ in patients with dementia stratified by disease severity.** Panels show the statistical comparison performed using the Kruskal-Wallis test. Significant *p* values are reported above each comparison. The legend indicates the color coding corresponding to the different dementia subgroups. Statistical significance was defined as p < 0.05.

**Figure S14**. **Quantitative PCR (qPCR) analysis of HERV-W gene expression in patients with dementia, stratified by disease severity.** Panels show the statistical comparison performed using the Kruskal-Wallis test. Significant *p* values are reported above each comparison. The legend indicates the color coding corresponding to the different dementia subgroups. Statistical significance was defined as p < 0.05.

**Figure S15**. **Analysis of antibody levels against HERV-H_(229-241)_ in patients with dementia stratified by disease severity.** Panels show the statistical comparison performed using the Kruskal-Wallis test. No statistically significant *p* values were observed. The legend indicates the color coding corresponding to the different dementia subgroups. Statistical significance was defined as p < 0.05.


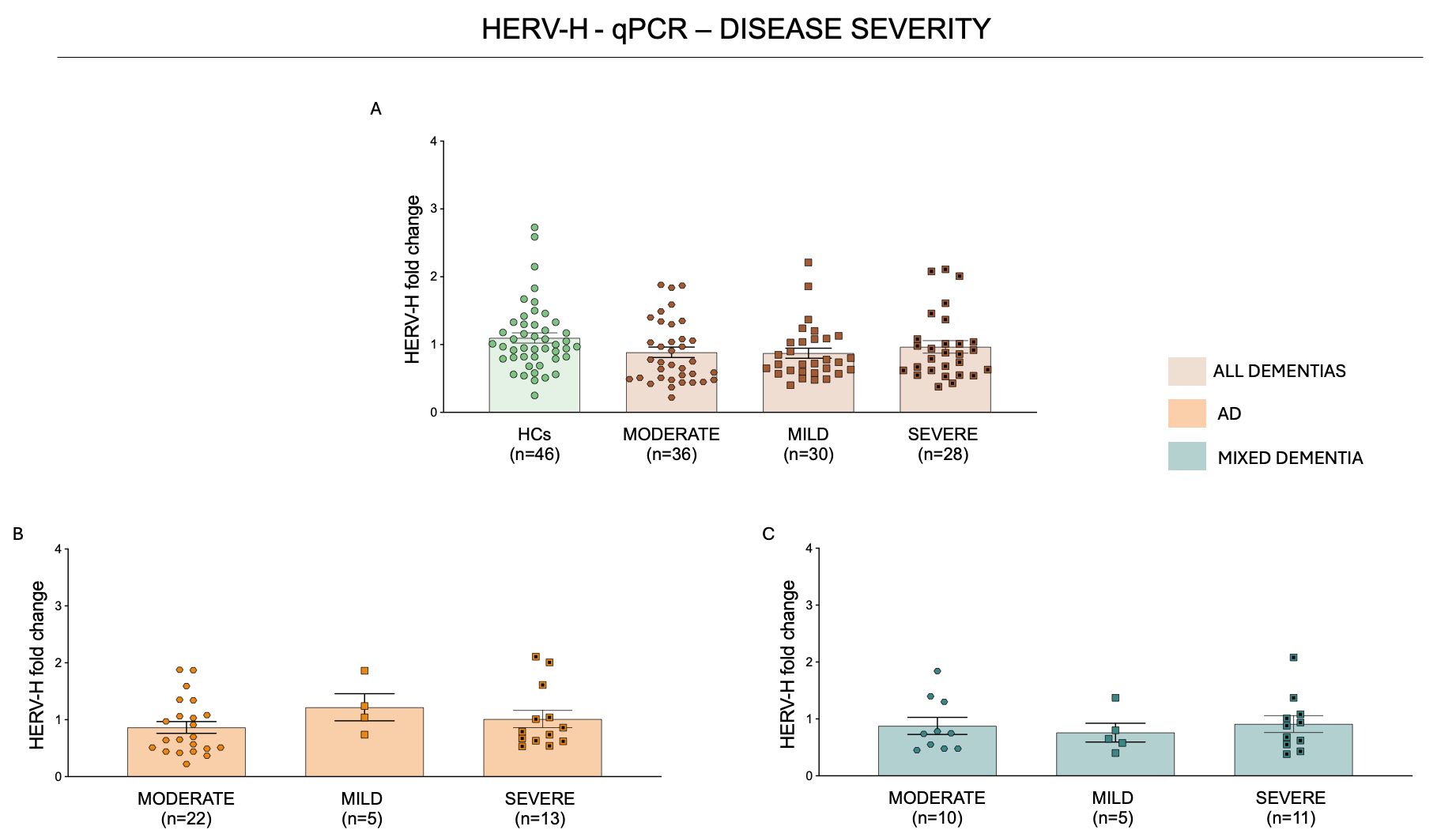


**Figure S16**. **Quantitative PCR (qPCR) analysis of HERV-H gene expression in patients with dementia, stratified by disease severity.** Panels show the statistical comparison performed using the Kruskal-Wallis test. No statistically significant *p* values were observed. The legend indicates the color coding corresponding to the different dementia subgroups. Statistical significance was defined as p < 0.05.

**Figure S17**. **Analysis of antibody levels against ASRGL1_(14-25)_ in patients with dementia stratified by disease severity.** Panels show the statistical comparison performed using the Kruskal-Wallis test. Significant *p* values are reported above each comparison. The legend indicates the color coding corresponding to the different dementia subgroups. Statistical significance was defined as p < 0.05.

**Figure S18**. **Quantitative PCR (qPCR) analysis of ASRGL1 gene expression in patients with dementia, stratified by disease severity.** Panels show the statistical comparison performed using the Kruskal-Wallis test. No statistically significant *p* values were observed. The legend indicates the color coding corresponding to the different dementia subgroups. Statistical significance was defined as p < 0.05.

**Figure S19. Antibody responses and gene expression of HERV-W, HERV-H, and ASRGL1 in patients with dementia stratified by disease duration.** Analysis of antibody levels against HERV-W_(248-262)_ (A), HERV-H_(229-241)_ (C), and ASRGL1_(14-25)_ (E) and, gene expression of HERV-W (B), HERV-H (D) and ASRGL1 (F) in patients with dementia, stratified by disease duration. Panels show the statistical comparison performed using the Kruskal-Wallis test. Significant *p* values are reported above each comparison. Statistical significance was defined as p < 0.05.

**Figure S20. Antibody responses and gene expression of HERV-W, HERV-H, and ASRGL1 in patients with dementia stratified by age.** Analysis of antibody levels against HERV-W_(248-262)_ (A), HERV-H_(229-241)_ (C), and ASRGL1_(14-25)_ (E) and, gene expression of HERV-W (B), HERV-H (D) and ASRGL1 (F) in patients with dementia, stratified by disease duration. Panels show the statistical comparison performed using the Kruskal-Wallis test. Significant *p* values are reported above each comparison. Statistical significance was defined as p < 0.05.
